# Hypoxia-inducible factor-1 alpha expression is induced by IL-2 via the PI3K/mTOR pathway in hypoxic NK cells and supports effector functions in NKL cells and ex vivo expanded NK cells

**DOI:** 10.1007/s00262-021-03126-9

**Published:** 2022-01-09

**Authors:** Emily Cluff, Carina C. Magdaleno, Emyly Fernandez, Trenton House, Srividya Swaminathan, Archana Varadaraj, Narendiran Rajasekaran

**Affiliations:** 1 Department of Chemistry and Biochemistry, Northern Arizona University, 700 S Osbourne Drive, Flagstaff, AZ 86004 USA; 2 Department of Systems Biology, Beckman Research Institute of City of Hope, Duarte, CA 91010 USA

**Keywords:** Hypoxia, Natural killer cells, IL-2, HIF-1α

## Abstract

Natural killer (NK) cells are cytotoxic innate lymphocytes that are specialized to kill tumor cells. NK cells are responsive to the primary cytokine IL-2 in the tumor microenvironment (TME), to activate its effector functions against tumors. Despite their inherent ability to kill tumor cells, dysfunctional NK cells observed within advanced solid tumors are associated with poor patient survival. Hypoxia in the TME is a major contributor to immune evasion in solid tumors that could contribute to impaired NK cell function. HIF-1α is a nodal regulator of hypoxia in driving the adaptive cellular responses to changes in oxygen concentrations. Whether HIF-1α is expressed in hypoxic NK cells in the context of IL-2 and whether its expression regulates NK cell effector function are unclear. Here, we report that freshly isolated NK cells from human peripheral blood in hypoxia could not stabilize HIF-1α protein coincident with impaired anti-tumor cytotoxicity. However, ex vivo expansion of these cells restored HIF-1α levels in hypoxia to promote antitumor cytotoxic functions. Similarly, the human NK cell line NKL expressed HIF-1α upon IL-2 stimulation in hypoxia and exhibited improved anti-tumor cytotoxicity and IFN-γ secretion. We found that ex vivo expanded human NK cells and NKL cells required the concerted activation of PI3K/mTOR pathway initiated by IL-2 signaling in combination with hypoxia for HIF-1α stabilization. These findings highlight that HIF-1α stabilization in hypoxia maximizes NK cell effector function and raises the prospect of NK cells as ideal therapeutic candidates for solid tumors.

**Supplementary Information:**

The online version contains supplementary material available at 10.1007/s00262-021-03126-9.

## Introduction

Natural killer cells (NK), the major innate effector cells, with their broad cytotoxicity against tumors are ideal candidates for immunotherapy [1, 2]. NK cells are highly efficient in killing tumor cells since they do not require any priming or prior activation, unlike cytotoxic T cells, which rely on priming by antigen presenting cells. On encountering a tumor cell, the NK cell’s ability to kill is determined by the integration of signals from activating and inhibitory receptors expressed on their surface [1]. In addition to expression of ligands to these receptors on tumor cells, oxygen deprivation or hypoxia, universally associated with the tumor microenvironment (TME), is also a key determinant of the NK cell cytotoxic response. Hypoxia is considered an adverse prognostic factor for solid tumor regression and can be detrimental to antitumor effector immune cell function [3, 4]. NK cells have been previously shown to exhibit impaired cytolytic functions under hypoxic conditions [2]. Given this, NK cells must initiate dynamic mechanisms to adapt to changes in oxygen tension to execute their functions. Adaptations to hypoxia are facilitated by increased expression and stabilization of transcription factor hypoxia-inducible factor-1 (HIF-1), which mediates increased transcriptional activation, glycolysis, proliferation, and angiogenesis [9]. The underlying factors in the TME that leads to HIF upregulation in NK cells and the consequence of this upregulation to NK cell responses during hypoxia have not been thoroughly investigated.

HIF-1 is a basic heterodimeric helix–loop–helix PAS (PER/ARNT/SIM), consisting of a HIF-1α subunit complexed to the HIF-1β subunit. HIF-1α is transcribed and synthesized in response to signals from growth factors and ligands [3]. Importantly, HIF-1α is sensitive to changes in cellular oxygen levels and is therefore regarded as a central player in the hypoxic response of the cell. Under normal oxygen tension (normoxia), the critical proline residues on HIF-1α subunits are hydroxylated (P402 and P564), recognized by pVHL, poly-ubiquitinated, and subsequently degraded via the ubiquitin–proteasome pathway [4]. However, in hypoxic conditions, HIF-1α subunits are expressed but cannot be prolyl hydroxylated and they escape VHL recognition and subsequent degradation. The HIF-1α subunits that accumulate heterodimerize with HIF-1β to activate target gene transcription. In immune cells such as T cells and macrophages, HIF-1α is induced both by hypoxic and non-hypoxic stimuli such as T cell receptor signaling and PI3Kinase (PI3K)-mediated pathways [5–8].

The common cytokine-receptor gamma-chain cytokine IL-2 is a prevalent cytokine in the TME and activates NK cells through the PI3K pathway. IL-2 modulates the immune landscape of tumors and is essential for NK cell activation and function [9–12]. Importantly, soluble IL-2 secreted by activated T helper cells in response to antigen presentation in the TME can activate NK cells expressing high and intermediate affinity receptors [13]. Addressing the response of NK cells to hypoxia in the context of IL-2 stimulation is imperative to the development of NK cells as better candidates for immunotherapy.

Here, we explored the role of IL-2 in HIF-1α expression in hypoxic NK cells. Using the NK cell line, NKL, we established that IL-2 initiates HIF-1α protein synthesis through the PI3K/mTOR pathway, while hypoxia stabilizes the HIF-1α protein. We then sought to extend our findings to human peripheral blood mononuclear cell (PBMC)-derived NK cells. We observed that freshly isolated NK cells do not stabilize HIF-1α in response to hypoxia, whereas ex vivo expansion of these cells demonstrated detectable levels of HIF-1α in response to hypoxia. In addition, we report here that HIF-1α expression in IL-2 stimulated hypoxic NK cells correlated with enhanced cytotoxic and cytokine-secreting functions.

## Materials and methods

### Cell lines and culture

The NK cell line, NKL, was a kind gift from Prof. Lewis Lanier, UCSF. NKL cells were cultured in RPMI 1640 medium from Corning supplemented with 10% heat-inactivated FBS, 5% non-essential amino acids, 5% sodium pyruvate, 5% penicillin streptomycin, and 100 U/mL IL-2. The colorectal cancer cell line, DLD-1, was purchased from ATCC (#CCL-221) and was cultured in RPMI 1640 medium from Corning supplemented with 10% heat-inactivated FBS and 5% penicillin streptomycin. Cell lines were maintained in normoxic conditions at 21% O_2_ in a 37 °C humidified incubator buffered with 5% CO_2._ For hypoxic conditions, NK cells were incubated in a hypoxic chamber (Biospherix, C-Chamber Incubator Subchamber) with a mixture of 1% O_2_, 5% CO_2_, and 94% N_2_ placed in a 37 °C humidified incubator for 24 h (NKL cells) or 72 h (PBMC NK cells). After incubation, NK cells were immediately transferred to conical tubes pre-cooled on ice and spun down at 4 °C for further processing.

### Peripheral blood NK cell isolation and expansion

Peripheral blood was purchased as leukoreduction system chamber buffy coats from healthy donors at the Stanford Blood Center, Stanford. PBMCs were isolated by Ficoll-Paque PLUS (GE Healthcare) density gradient centrifugation in RPMI 1640 medium from Corning. NK cells were enriched from the PBMCs by negative selection using the NK cell isolation kit, human (Miltenyi Biotec GmbH, Bergisch Gladbach, Germany, catalog #130-092-657), according to the manufacturer's instructions by using a LS column (Miltenyi Biotec GmbH, #130-042-401) on the Midi MACS separator (Miltenyi Biotec GmbH, #130-042302).

NK cell expansion was achieved by using the “NK cell activation/expansion kit” (Miltenyi Biotec GmbH, catalog #130-094-483) according to the manufacturer's instructions. Briefly, NKp46 and CD2 biotinylated antibodies were loaded on anti-biotin-MACSiBead™ particles. MACSiBead™ particles loaded with the biotinylated antibodies were added to NK cells and cultured for 2 weeks, in NK MACS medium (Miltenyi Biotec GmbH, #130-114-429), with 5% human AB serum (Sigma–Aldrich), 1% supplement (Miltenyi Biotec GmbH, #130-114-429), 1% penicillin–streptomycin, and 400 U/mL IL-2 in 24-well plates. NK cells were passaged every 3 days at a cell density of 1–1.5 × 10^6^ NK cells per mL of media. NK cells were maintained in a 37 °C humidified incubator buffered with 5% CO_2_. Purity of isolated NK cells was determined by flow cytometry and preparations stained ≥ 97% CD56^+^CD3^−^ with ≤ 1% of CD3^+^, CD14^+^, and CD20^+^ cells.

### Flow cytometry

Expression of activation and inhibitory receptors on NK cells was determined by flow cytometry. Before cell surface staining, Fc receptors on NK cells were blocked with human TruStain FcX from BioLegend (#422301). Cells (∼0.5 × 10^6^) were then stained with viability dye (LIVE/DEAD Fixable Dead Cell Stain, ThermoFisher, #L34955), followed by staining with cell surface antibodies (listed in Antibodies and Reagents) or the corresponding isotype in 100 µl volume for 30 min on ice, followed by washes in FACS buffer (PBS with 0.25% BSA and 1 mM EDTA). A fluorescence-minus-one (FMO) control was used in all experiments to determine negative staining. Cells were subsequently fixed in BD Cytofix Fixation Buffer (BD Biosciences, #BDB554655) according to instructions. All samples were run on a CytoFlex flow cytometer (Beckman coulter) and analyzed using FlowJo v10.6.0 software.

### NK cell cytotoxicity assay

NK cell cytotoxicity against target tumor cells was determined by the xCELLigence RTCA (Real-Time Cell Analyzer) SP (single-plate) Instrument (Agilent, San Diego, CA, USA) according to manufacturer’s recommendations. Fifty microliters of complete medium was added to each well of the E-plate 96, and background impedance on the plates was measured on the xCELLigence RTCA SP instrument at 37° and 5% CO_2_. DLD-1 cells (1 × 10^4^/well) were seeded in the plate and used as target cells. DLD-1 cells are derived from human colorectal adenocarcinoma and exhibit mutations in their KRAS gene. They lack the surface expression of MHC class I and are thus highly susceptible to NK cell-mediated cytotoxicity. The E-plate was then placed in the xCELLigence RTCA SP cradle, and impedance measurements were recorded every 15 min. After 24 h, NK cells at various effector-to-target ratios were added in plates as effector cells. For hypoxia experiments, NKL cells were incubated in 1% O_2_ for 24 h and PBMC NK cells for 72 h prior to being added to the target cells. Impedance measurements were recorded every 15 min, and death of tumor cells was indicated by a decrease in cell index. Data were acquired with RTCA Software 1.2 (Agilent, Biosciences). Samples were internally normalized for the cell index value measured before NK cell addition (normalized cell index). The normalized cell index is converted to a % cytolysis plot by the xCELLigence Immunotherapy Software (xIMT).

### Western blotting

Immunodetection of proteins was performed as described previously [14]. For preparation of whole-cell lysates, NK cells were lysed in ice-cold SDS lysis buffer containing protease and phosphatase inhibitors (1 mM EDTA, 1 mM PMSF, 1 mM DTT, 1 µg/mL leupeptin, 1 mM sodium orthovanadate), followed by sonication and centrifugation at 300 g for 10 min. Lysates were separated by SDS–PAGE and immunoblotted for specific proteins as indicated. β-Actin or tubulin was used as loading control. Blots were imaged using the Odyssey Imaging System (LI-COR Biosciences). Quantification of immunoblots was performed using the Li-Cor Image Studio Software, version 5.2. Pixel intensities for each protein were measured and normalized to the loading control. Average normalized pixel intensities from multiple independent experiments were determined, and fold differences between untreated and treated samples were plotted. *P*-values were calculated using the unpaired Student’s t-test.

### RNA extraction and qPCR analysis

Total RNA was extracted from NKL cells using the Qiagen RNeasy kit (#74104) according to manufacturer’s protocol. RNA quality was assessed using the Nanodrop, and 550 ng RNA was used to create cDNA using the TaqMan Reverse Transcription kit (N808-0234). Ten nanograms of cDNA was used in duplicate qPCRs using master mix from TaqMan Universal PCR Master Mix (#4324018) and TaqMan primer probes. The HIF-1α, #HS00153153_M1 primer probes that span exons were used to quantify mRNA levels. qPCR was performed on an Applied Biosystems 7500 SDS real-time PCR system. β-Actin primer probes were used for normalization. Relative expression was calculated using the delta Ct method.

### ELISA

NK cells were plated 80,000 cells per 200 µL in a 96-well plate with 200 U/mL IL-2 and pre-incubated in 21% or 1% O_2_ for 24 h. Corning 96-Well TC-Treated Microplates were coated with 10 µg/mL of each antibody/ligand (Recombinant Human ULBP-1, R &D Systems #1380-UL-050; UltraLeaf anti-CD16 ab (3G8), Biolegend #302057; UltraLeaf anti-NKp46 ab (9E2), Biolegend #331947) for 3 h and then washed three times with 1 × PBS. After antibody/ligand coating of the plates, the NK cells were then transferred to the coated plate and incubated for 18 h in 21% or 1% O_2_. After incubation, cells were spun down at 300 × g for 5 min. Supernatant was removed and stored at -20 °C until ELISA was performed. Human IFN-γ was assayed with the human IFN-gamma DuoSet ELISA kit (R&D DY285B) and granzyme B was assayed with human granzyme B DuoSet ELISA kit (R&D DY2906-05) according to manufacturer’s instructions. Concentrations were drawn from a standard curve performed on each plate.

### Antibodies and reagents

The mAb against HIF-1α (#14179), p70 S6 kinase (#9202), and phosphorylated p70 S6 kinase at Thr389 (#9205) was purchased from Cell Signaling Technology. β-Actin (#MA1-140) was purchased from Invitrogen. Abs against ERK1–ERK2 (#ab17942) and phosphorylated ERK1–ERK2 at T202 and Y204 (#ab214362) were purchased from Abcam. Recombinant human IL-2 (#200-02) was obtained from PeproTech. The details of antibodies used in this study are listed in supplementary table 1. Inhibitors of PI3K (LY294002, #S1105), MEK (PD98059, #S1177), and the mammalian target of rapamycin (rapamycin, #AY-22989) were purchased from Selleck Chemicals. DMOG (#ab141586) was purchased from Abcam. Antibodies used for flow cytometry were purchased from BioLegend: PE-conjugated anti-NKp46 (#137603), Percp.cy5.5-conjugated anti-NKG2D (#320818), FITC-conjugated anti-CD69 (#310903), APC-conjugated anti-CD56 (#318310), and Alexa Fluor 700-conjugated anti-CD16 (#302026).

### Statistical analyses and quantification

The Prism 9 (GraphPad) software was used for all statistical analyses. Comparisons between hypoxia-treated and normoxia-treated samples were made using Student’s *t*-test. Statistical test for each figure is described in the figure legend. qPCR assays were run in technical and biological replicates. All other assays were performed as multiple independent trials and represented as mean ± SEM. Numbers of independent experiments (n) are given in the figure legends. For western blots, one out of three independent experiments is shown. A *p* ≤ 0.05 was considered significant.

## Results

### HIF-1α upregulation in hypoxic NK cells requires IL-2 stimulation

**Fig. 1:**
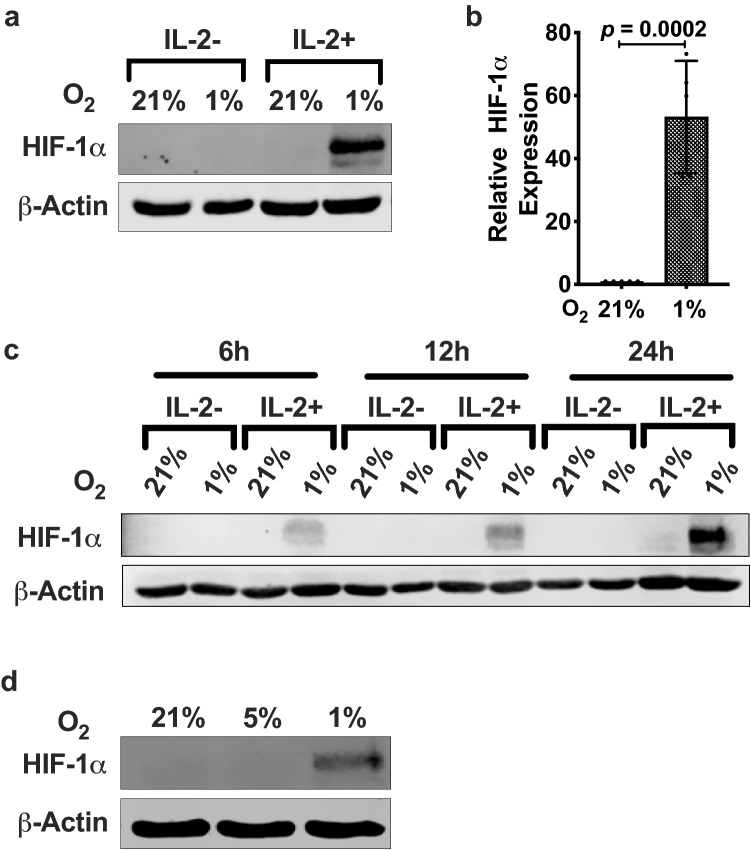
IL-2-induced HIF-1α protein expression in hypoxic NKL cells. **a** Immunoblot of HIF-1α expression. NKL cells were incubated in the absence or presence of 100 U/mL IL-2 for 24 h at 21% or 1% O_2_. Cells were lysed and immunoblotted for HIF-1α. β-Actin was used as the loading control. Blot is representative of > 4 independent trials. **b** Quantification of immunoblots presented as bar graphs are averages of four independent trials ± SEM. Statistical significance and *p* values were determined using the unpaired two-tailed Student’s *t*-test. *p ≤ *0.05 is significant. **c** Time-dependent increase in HIF-1α protein levels. NKL cells were incubated in the absence or presence of 100 U/mL IL-2 for the indicated times at 21% or 1% O_2_. Cell lysates were immunoblotted for HIF-1α and β-Actin was used as the loading control. **d** Effect of physioxia (5% O_2_) on HIF-1α protein expression. IL-2-stimulated NKL cells exposed to 21%, 5%, or 1% O_2_ for 6 h were lysed and immunoblotted for HIF-1α. Data are representative of two independent trials

To investigate whether NK cells upregulate HIF-1α in response to hypoxia, we exposed the human NK cell line, NKL, cultured in the presence of IL-2 to hypoxic (1% O_2_) or normoxic (21% O_2_) culture conditions. Under normoxia, we observed no detectable HIF-1α protein by immunoblotting (Fig. 1a). Exposure to hypoxia for 6 h significantly increased HIF-1α protein levels (*p* = 0.0002) (Fig. 1a and b). NKL cells in hypoxia and deprived of IL-2 did not show HIF-1α protein. Upon increasing the time of exposure of NKL cells to hypoxia to 12 h and 24 h, we observed a time-dependent increase in HIF-1α protein levels (Fig. 1c). However, NKL cells deprived of IL-2 did not show detectable HIF-1α protein even after 24 h of exposure to hypoxia (Fig. 1c). Since physiological oxygen concentration (physioxia) in secondary lymphoid organs is around 5% O_2_, we investigated whether physioxia has an effect on HIF-1α levels. In NKL cells exposed to 5% O_2_ and cultured in the presence of IL-2, we observed no HIF-1α protein (Fig. 1d). These data conclusively establish that the NK cell line NKL expresses HIF-1α under hypoxic conditions but only in the presence of IL-2 stimulation.

### IL-2 increases HIF-1α translation in NK cells

**Fig. 2:**
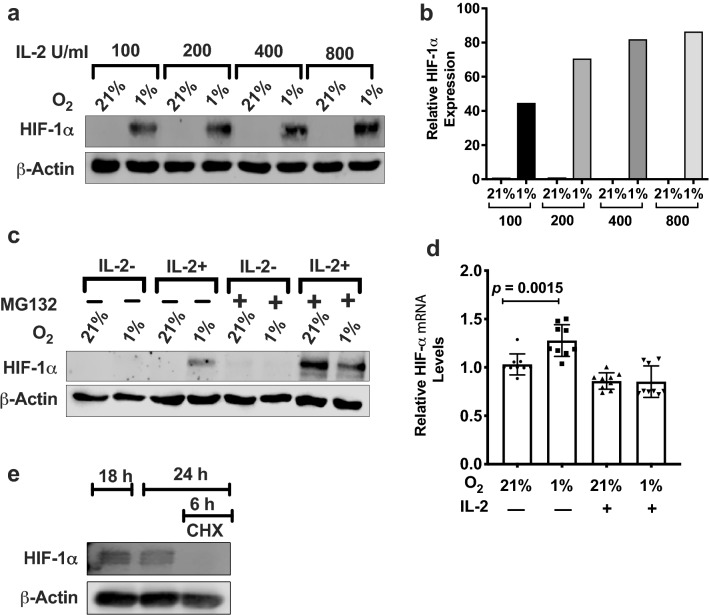
HIF-1α protein expression and stabilization in hypoxic NKL cells**. a** NKL cells were incubated in the presence or absence of the indicated concentrations of IL-2 for 24 h at 21% or 1% O_2_ and HIF-1α protein expression detected by immunoblotting. β-Actin was used as the loading control**. b** Quantification of immunoblots presented as bar graphs. Density of bands was normalized to β-actin and shown as fold difference of HIF-1α protein expression in hypoxic NK cells compared to 21% O_2_ with 100 U/mL IL-2. Blot is representative of two independent trials. **c** Immunoblot of HIF-1α protein in NKL cells incubated with or without 100 U/mL IL-2 in the presence or absence of 10 μg/mL of MG132 for 6 h at 21% or 1% O_2_. Blot is representative of three independent trials. **d** qPCR analysis for HIF-1α mRNA**.** NKL cells were incubated in the presence or absence of 100 U/mL IL-2 for 24 h at 21% or 1% O_2_ and cells processed for RNA extraction. Relative expression of HIF-1α mRNA was determined using the delta Ct method. β-Actin was used for normalization. Data are an average of three independent trials each performed in triplicates. Statistical significance and *p* values were determined using the unpaired Student’s *t*-test. *p* ≤ 0.05 is significant. **e** Immunoblot of HIF-1α protein levels in NKL cells incubated for 18 h in 1% O_2_ and then incubated in the presence or absence of 10 μg/mL CHX for an additional 6 h in 1% O_2_. β-Actin was used as loading control. Blot is representative of three independent trials

HIF-1α expression in cells is regulated at multiple levels, with hypoxia inhibiting its degradation and cell specific upstream signals regulating its translation and transcription. To delineate the contribution of hypoxia from IL-2 in increasing HIF-1α levels, we first tested whether HIF-1α expression is dependent on the concentration of IL-2. NKL cells cultured in hypoxia when treated with increasing concentrations of IL-2 showed a corresponding increase in HIF-1α levels (Fig. 2a and b). However, NKL cells under normoxia showed no detectable levels of HIF-1α even when IL-2 concentration in the media was increased eightfold. These results indicate that IL-2 stimulation induced the synthesis of HIF-1α protein but its stabilization was dependent on hypoxia.

Since hypoxia prevents proteasomal degradation and stabilizes HIF-1α in multiple cell types, we investigated whether proteasomal degradation similarly regulates HIF-1α stabilization in NK cells. NKL cells treated with the proteasome inhibitor MG132 in the presence of IL-2 stimulation restored HIF-1α proteins levels under normoxia (Fig. 2c; lanes 3 and 7). However, inhibiting proteasomal degradation in the absence of IL-2 did not restore HIF-1α protein under either normoxia or hypoxia, confirming that HIF-1α protein cannot be synthesized in the absence of IL-2 and that hypoxia by itself was unable to induce any HIF-1α protein (Fig. 2c; lanes 1, 2, 5, and 6). Thus, IL-2 induced the synthesis of HIF-1α protein in NK cells that is degraded by the proteasome under normoxia, while exposure to hypoxia stabilized the HIF-1α protein from being degraded.

We next addressed whether IL-2 exerted transcriptional or translational control of HIF-1α protein synthesis. To find whether IL-2 increased HIF-1α protein synthesis by increasing HIF-1α transcription, we quantified HIF-1α mRNA in NKL cells cultured under normoxia and hypoxia with or without IL-2. Though in the absence of IL-2 we observed an increase in HIF-1α transcript levels in hypoxia, IL-2 stimulation did not significantly increase HIF-1α transcript levels in NKL cells in either normoxia or hypoxia conditions. This indicates that the increase in HIF-1α protein synthesis observed in NKL cells is not a consequence of IL-2-driven HIF-1α transcription (Fig. 2d). For testing the role of translation in HIF-1α protein synthesis, NKL cells cultured in the presence of IL-2 and hypoxia for 18 h were then additionally treated with the translation inhibitor cycloheximide (CHX) for another 6 h under hypoxia. CHX treatment completely abrogated HIF-1α protein expression compared to the untreated cells, demonstrating that IL-2 mediated de novo HIF-1α protein translation (Fig. 2e).

### HIF-1α protein is upregulated via PI3K/mTOR signaling pathway under hypoxic conditions in NK cells

**Fig. 3:**
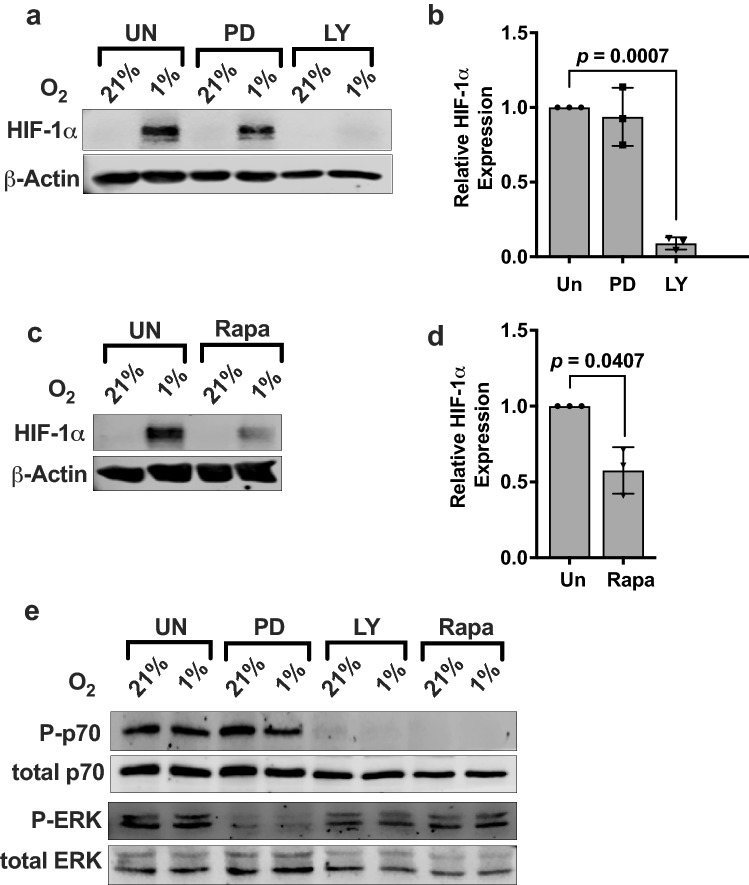
IL-2 mediates HIF-1α expression in NKL cells through the PI3K/mTOR signaling pathway. **a** IL-2-stimulated NKL cells were incubated for 24 h at 21% or 1% O_2_ in the presence or absence of 50 μM PD98059 (MAPK inhibitor) or 50 μM LY294002 (PI3K inhibitor). Lysates were immunoblotted for HIF-1α. β-Actin is the loading control. **b** Quantification of immunoblots normalized to β-actin is presented as fold difference of HIF-1α levels in 1% O_2_ relative to inhibited samples. Bar graph is representative of three independent experiments. **c** IL-2-stimulated NKL cells were incubated for 24 h at 21% or 1% O_2_ in the presence or absence of 10 nM rapamycin. Lysates were immunoblotted for HIF-1α. β-Actin is the loading control. **d** Quantification of immunoblots normalized to β-Actin is presented as fold difference of HIF-1α levels in 1% O_2_ relative to the inhibited sample. Bar graph is representative of three independent experiments. Statistical significance and *p* value between inhibitor treated and uninhibited were determined by unpaired two-tailed Student’s *t*-test. *p* ≤ 0.05 is significant. **e** IL-2-stimulated NKL cells exposed to 21% or 1% O_2_ were untreated or treated with 50 μM PD98059, 50 μM LY294002, or 10 nM rapamycin. Lysates were immunoblotted for the proteins as shown

Activation and survival of NK cells are promoted by the binding of IL-2 to its receptor, leading to signaling via the phosphoinositide 3-kinase (PI3K) and mitogen-activated protein kinase (MAPK) pathways [15, 16]. To investigate whether these signaling mechanisms also drive HIF-1α protein synthesis, we blocked IL-2 signaling with the pan-PI3K inhibitor LY294002 and MAPK inhibitor PD98059. Under hypoxic conditions, LY294002 treatment significantly reduced HIF-1α protein levels in IL-2-stimulated NKL cells under hypoxia (*p* = 0.0007), while remaining unaffected by PD98059 (Fig. 3a and b), demonstrating that IL-2 signaling via the PI3K pathway and not through the MAPK pathway is required for HIF-1α expression. Since mTORC1 is the main kinase downstream of the PI3K pathway, we determined whether HIF-1α protein synthesis is sensitive to the mTORC1 inhibitor rapamycin. Rapamycin significantly reduced hypoxia-induced HIF-1α protein expression in IL-2-stimulated NK cells (*p* = 0.0407) (Fig. 3c and d). The specificity of these inhibitors in blocking their targeted signaling pathways in NKL cells was confirmed by immunoblotting for phosphorylation of kinases downstream of PI3K and MAPK, p70S6K and P-Erk (Fig. 3e). As shown, hypoxia did not affect the phosphorylation or protein levels of these downstream kinases, indicating that low oxygen concentration did not modulate these signaling pathways in NKL cells. These results add credence to our observation that IL-2 activation of PI3K/mTOR signaling cascade in NK cells induces HIF-1α expression.

### NKL cells exhibit increased anti-tumor cytotoxicity and IFN-γ secretion in hypoxia

**Fig. 4:**
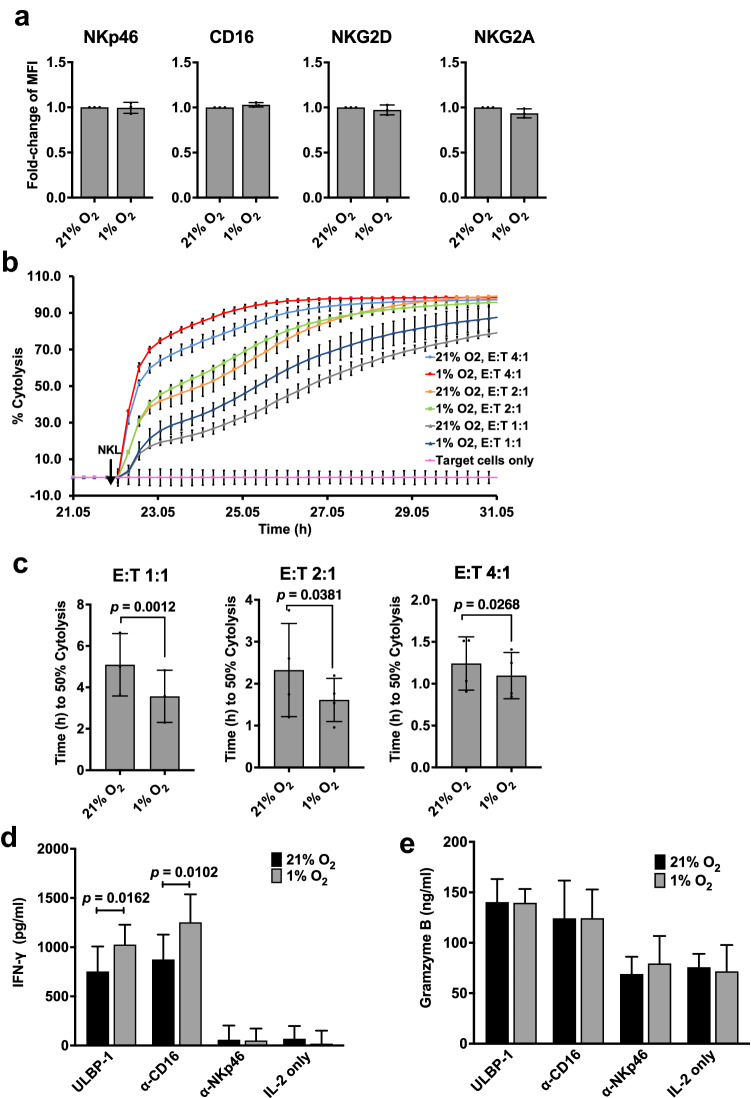
NKL cells in hypoxia exhibit increased cytolytic activity and IFN-γ secretion. **a** Characterization of NK cell receptors on NKL cells in hypoxia. NKL cells in the presence of IL-2 were exposed to 21% or 1% O_2_ for 24 h. Expression of surface receptors NKG2D, NKp46, CD16 and NKG2A was analyzed by flow cytometry as described in “Materials and methods.” Fold changes in median fluorescence intensity (MFI) ± SEM of hypoxic NKL cells against normoxic NKL cells are shown (*n* = 3). Statistical significance was analyzed between normoxia and hypoxia by a paired two-tailed Student’s *t*-test. **b** Real-time analysis of cytolysis exhibited by hypoxia-treated NKL cells against target tumor cells. At 24 h after seeding the target DLD-1 cells, NKL cells pre-incubated in hypoxia or normoxia in the presence of IL-2 for 24 h were added to the target cells at E/T ratios of 4:1, 2:1, and 1:1. Real-time cytolysis of target cells was monitored using the xCELLigence RTCA SP system. Electrode impedance was measured and recorded as cell index. % Cytolysis was then determined using the RTCA Software Pro. One representative of three independent experiments performed in duplicates is shown. **c** 50% killing time (KT 50) for the same E/T ratios in **b**. **d** NKL cells incubated at 21% or 1% O_2_ for 24 h in the presence of IL-2 before being transferred to plates coated with ULBP-1 or anti-NKp46 or anti-CD16 antibodies, and incubations continued under same conditions for an additional 18 h. After incubation, supernatant was collected and ELISA performed to test the concentrations of IFN-γ and granzyme B. Results are reported as the mean of four independent experiments. Statistical significance was analyzed between 21% O_2_ and 1% O_2_ using two-tailed paired Student’s *t*-test. *p* ≤ 0.05 is significant

Since we observed a hypoxia-induced upregulation of HIF-1α expression in NKL cells, we next investigated whether this was associated with any phenotypic and functional changes in NKL cells. NK cells express both inhibitory and activating receptors that modulate its functions upon ligand binding. Studying the expression of the receptors on NK cell surface would indicate their activated or inhibited status upon hypoxia exposure. NKL cells exposed to hypoxia for 24 h were subsequently analyzed for the surface expression of the inhibitory receptor, CD94/NKG2A heterodimer that mediates a strong inhibition of NK cell cytotoxicity. We also tested the activating receptors NKG2D, NKp46, and CD16 by flow cytometry. As shown in Fig. 4a and supplementary Fig. 1, the expression of these receptors on NK cells remained comparable under normoxia and hypoxia.

We next tested whether hypoxia has any effect on the cytolytic functions of NKL cells. To test this, we used the impedance-based real-time quantification of cell death of tumor cells upon NK cell addition. In traditional end-point cytotoxicity assays, both the NK cells (effector cells) and the tumor cells (target cells) would be co-incubated for a period of time in either hypoxia or normoxia. Upon using that approach, it is difficult to interpret whether the contribution of hypoxia to NK cell cytotoxicity is due to the NK cells under hypoxia or the target cell response to hypoxia. Here, we used the impedance-based real-time assay to detect NK cell cytotoxicity where NK cells pre-exposed to hypoxia were co-cultured with normoxic tumor cells. Cytotoxicity measurements begin as early as 15 min upon NK cell addition in real time. This gave us the opportunity to examine the effect of hypoxia exclusively on the NK cells, by measuring real-time cytotoxicity exhibited by hypoxic NK cells immediately upon being added to tumor cells. NKL cells pre-incubated under hypoxia for 24 h were co-cultured with DLD-1 cells, at increasing effector-to-target (E/T) ratio, under normoxic conditions. NKL cell-mediated killing of DLD-1 cells was measured every 15 min and quantified as % cytolysis. Both NKL pre-incubated in hypoxia and NKL pre-incubated in normoxia demonstrated increase in cytolysis proportional to the increasing effector/target (E/T) ratios (Fig. 4b and c). However, NKL cells pre-incubated in hypoxia demonstrated a significantly higher cytotoxicity, requiring significantly less time to achieve 50% cytolysis of target DLD-1 cells when compared to NKL pre-incubated in normoxia (E/T 4:1, *p* = 0.0268; E/T 2:1, *p* = 0.0381; E/T 1:1, *p* = 0.0012) (Fig. 4b and c). Hypoxia-induced NK cytotoxicity was particularly high at the lower E/T ratios of 1:1 and 2:1 compared to the higher E/T ratio of 4:1.

We then compared the IFN-γ response of NKL cells in normoxic and hypoxic conditions. NKL treated with IL-2 and stimulated with ULBP1 or with anti-CD16 antibodies showed an increased expression of IFN-γ compared to unstimulated NKL cells (Fig. 4d). Interestingly, NKL cells stimulated with ULBP1 or with anti-CD16 antibodies in hypoxia showed a significant increase in IFN-γ secretion compared to NKL cells stimulated in normoxia (ULBP-1, *p* = 0.0162; anti-CD16 *p* = 0.0102) (Fig. 4d). However, stimulation with anti-NKp46 antibodies did not show any increase in IFN-γ irrespective of the oxygen concentrations. On testing for secretion of granzyme B by NKL cells, stimulation with ULBP1 or anti-CD16, but not with anti-NKp46 antibodies, resulted in an increase in granzyme B secretion and hypoxia failed to have any effect on its levels (Fig. 4e).

These results demonstrate that exposure of NKL cells to hypoxia increases their ability to kill tumor cells and also increases their ability to secrete IFN-γ.

### HIF-1α protein detection in human PBMC NK cells: HIF-1α expression in ex vivo expanded human NK cells is dependent on the PI3K/mTOR pathway

Data presented thus far illustrate the significance and requirement for IL-2 stimulation in HIF-1α expression during hypoxia by the NKL cells. We next sought to determine whether human PBMC-derived NK cells have a similar requirement for IL-2 for HIF-1α expression.

**Fig. 5:**
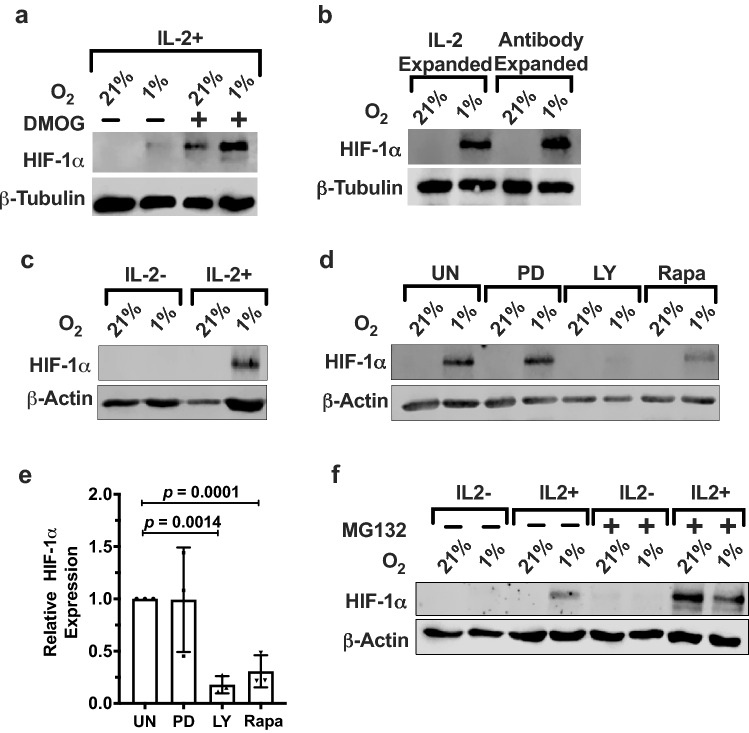
Ex vivo expanded PBMC NK cells express HIF-1α protein in the presence of IL-2. **a** Freshly isolated NK cells were prepared from human peripheral blood as described in “Materials and methods” and stimulated with 400 U/mL IL-2 and incubated for 72 h at 21% or 1% O_2_ in the presence or absence of 20 μM PHD inhibitor DMOG. HIF-1α and β-tubulin were detected by immunoblotting. Lanes lacking DMOG are representative of six different donors, and lanes with DMOG treatment are representative of three different donors. **b** PBMC-derived NK cells were expanded for 2 weeks using IL-2 only (IL-2 expanded) or IL-2 plus the Miltenyi MACSiBead™ loaded with anti-CD2 and anti-NKp46 abs (antibody expanded). NK cells were subsequently incubated with 400 U/mL IL-2 for 72 h at 21% or 1% O_2_. Whole-cell lysates were made and immunoblotted with anti-HIF-1α and β-tubulin antibodies. Blot is representative of seven different donors. **c** Ex vivo expanded NK cells were incubated in the absence or presence of 400 U/mL IL-2 for 72 h at 21% or 1% O_2_. Lysates were immunoblotted for HIF-1α. β-Actin is the loading control. **d** Ex vivo expanded NK cells were incubated with IL-2 for a total of 72 h at 21% or 1% O_2_ with the final 24 h in the presence of PD98059 (MAPK inhibitor), LY294002 (PI3K inhibitor), or rapamycin (mTOR inhibitor). Cell lysates were immunoblotted for HIF-1α. β-Actin is the loading control. Blot is representative of three independent experiments performed using three different donors. **e** Quantification of immunoblots in **d** normalized to β-actin and presented as fold difference of HIF-1α expression in 1% O_2_ relative to inhibited samples. Statistical significance was analyzed between inhibitor treated and control using paired two-tailed Student’s *t*-test. *p* ≤ 0.05 is significant. **f** Whole-cell lysates were prepared with ex vivo expanded NK cells incubated in the presence or absence of 400 U/mL of IL-2 and in the presence or absence of 10 µg/mL of MG132 for the final 24 h in a total 72-h incubation at 21% or 1% O_2_. HIF-1α and β-actin were detected by immunoblotting. Data are representative of two donors

Freshly isolated NK cells from human PBMCs were exposed to hypoxic or normoxic conditions for 72 h in the presence of IL-2. We observed no significant HIF-1α expression in NK cells exposed to hypoxia. Interestingly, upon treating freshly isolated NK cells with the prolyl hydroxylase (PHD) inhibitor DMOG for the duration of exposure to hypoxia, we observed detectable HIF-1α protein, indicating that HIF-1α was being synthesized in the freshly isolated NK cells but was being degraded even under low oxygen conditions (Fig. 5a). Since freshly isolated NK cells were not responding to hypoxia by expressing HIF-1α, we next tested whether ex vivo expanded NK cells are capable of expressing HIF-1α in response to hypoxia.

Ex vivo expanded NK cells show enhanced activation and higher cytotoxicity compared to freshly isolated NK cells and are promising candidates for adoptive cell therapy [17, 18]. We expanded NK cells by culturing them using the Miltenyi Biotec™, NK cell activation/expansion kit. In this method (antibody expansion), NK cells were cultured with anti-biotin microbeads loaded with a combination of biotinylated agonistic antibodies directed against NK cell-activating receptors NKp46 and CD2 and supplemented with IL-2. After two weeks of expansion, NK cells were subject to hypoxia for 72 h and tested for HIF-1α protein expression. Antibody-expanded NK cells showed robust HIF-1α protein expression under hypoxic conditions (Fig. 5b). Significantly, NK cells expanded just with IL-2 also expressed HIF-1α protein in hypoxia. Interestingly, there was no significant difference in the levels of HIF-1α protein between the two approaches used to expand PBMC NK cells (Fig. 5b). Importantly, following expansion, HIF-1α protein was no longer detected when IL-2 was depleted in these cells confirming the requirement of IL-2 for HIF-1α expression (Fig. 5c). We next tested whether the PI3K/mTOR pathway is required for HIF-1α upregulation in the ex vivo expanded NK cells. The antibody-expanded NK cells were treated with the PI3K inhibitor LY294002, the MEK inhibitor PD325901, and the mTOR inhibitor rapamycin and tested for HIF-1α protein levels. LY294002 and rapamycin treatment significantly reduced HIF-1α protein levels in the ex vivo expanded NK cells, while PD325901 had no effect (LY294002, *p* = 0.0014; rapamycin, *p* = 0.001) (Fig. 5d and e). These results demonstrate the dependence of the ex vivo expanded NK cells on the PI3K/mTOR pathway for HIF-1α expression. Furthermore, the ex vivo expanded NK cells treated with the proteasome inhibitor MG132 in the presence of IL-2 stimulation restored HIF-1α proteins levels under normoxia, indicating that the ex vivo expanded NK cells required hypoxia to stabilize HIF-1α protein (Fig. 5f).

The above results clearly demonstrate that freshly isolated NK cells from human PBMCs are unable to stabilize HIF-1α in response to hypoxia, while ex vivo expanded NK cells express and stabilize HIF-1α in an IL-2-dependent manner.

### Effect of hypoxia on PBMC-derived NK cell function

**Fig. 6:**
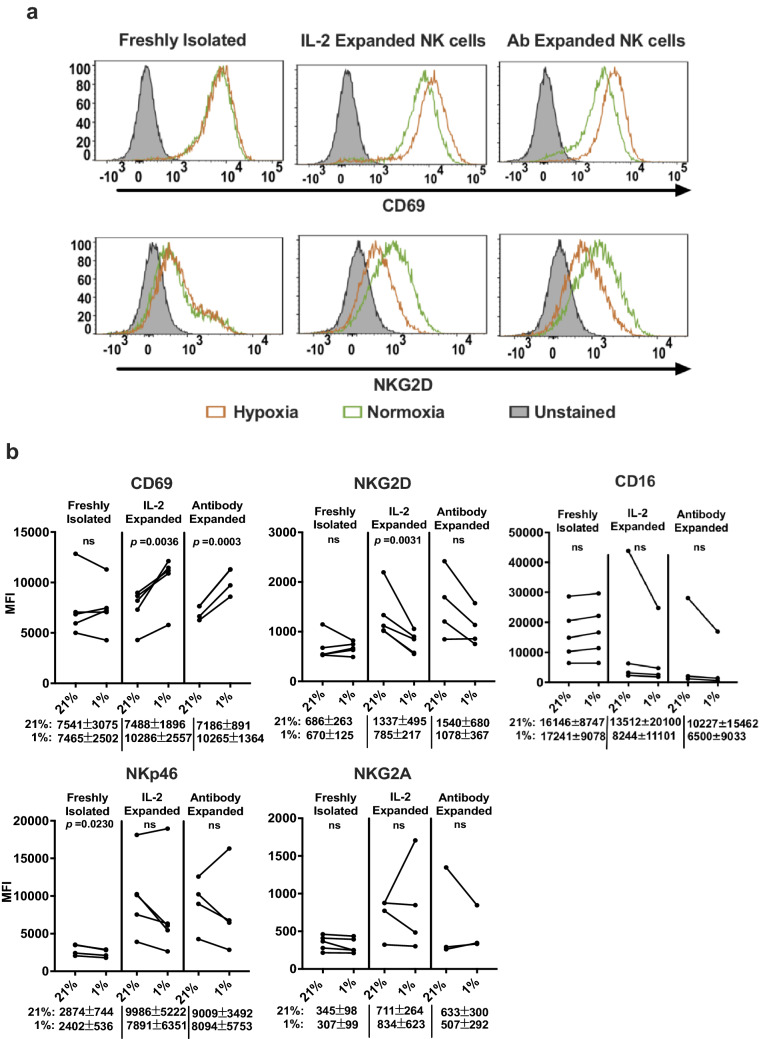
Characterization of NK cell receptors on PBMC-derived NK cells in hypoxia. Freshly isolated NK cells and ex vivo expanded NK cells were stimulated with IL-2 and incubated in 21% O_2_ or 1% O_2_ for 72 h. Expression of surface receptors was analyzed by flow cytometry as described in “Materials and methods.” **a** Representative histograms of CD69 and NKG2D expression on freshly isolated NK cells, IL-2-expanded NK cells, and antibody-expanded NK cells. **b** Median fluorescence intensity (MFI) of the surface receptors expressed on NK cells isolated from various donors is shown in the graph. The average median fluorescence intensities ± SEM of the surface receptors for each NK cell type exposed to normoxia or hypoxia are shown below the graphs. Statistical significance was analyzed between normoxia and hypoxia by a paired two-tailed Student’s *t*-test. *p* ≤ 0.05 is significant. NK cells isolated from a minimum of four donors were used for this analysis

We next examined the status of activating and inhibiting receptors on the PBMC-derived NK cells. Freshly isolated NK cells cultured in hypoxia for 72 h were subsequently analyzed for the surface expression of the inhibitory receptor, CD94/NKG2A, and activating receptors NKG2D, NKp46, CD16, and CD69 by flow cytometry. On comparing their expression under normoxia with hypoxia, hypoxia decreased expression of NKp46 that was significant *(p* = 0.0230). We observed no significant changes in the expression of NKG2D, CD69, or NKG2A (Fig. 6, supplementary Fig. 2). CD16 expression was slightly increased, but was not significant *(p* = 0.3189). On examining the ex vivo expanded NK cells, we observed that irrespective of whether they were expanded in IL-2 or antibody expanded, hypoxia resulted in the reduction in expression of NKG2D (IL-2 expanded, *p* = 0.0036; ab expanded, *p* = 0.0651), while CD69 was increased (IL-2 expanded, *p* = 0.0036; ab expanded, *p* = 0.0003). We did not see any significant change in NKG2A on either of the ex vivo expanded NK cells. NKp46 showed a reduction in expression in hypoxia, but was not significant (IL-2 expanded, *p* = 0.0517; ab expanded, *p* = 0.2709). This reduced expression of activation markers NKG2D and NKp46 that we observed on NK cells in hypoxia has also been reported in other studies [14]. However, here we report for the first time the increased expression of CD69 in expanded NK cells in hypoxia.

**Fig. 7:**
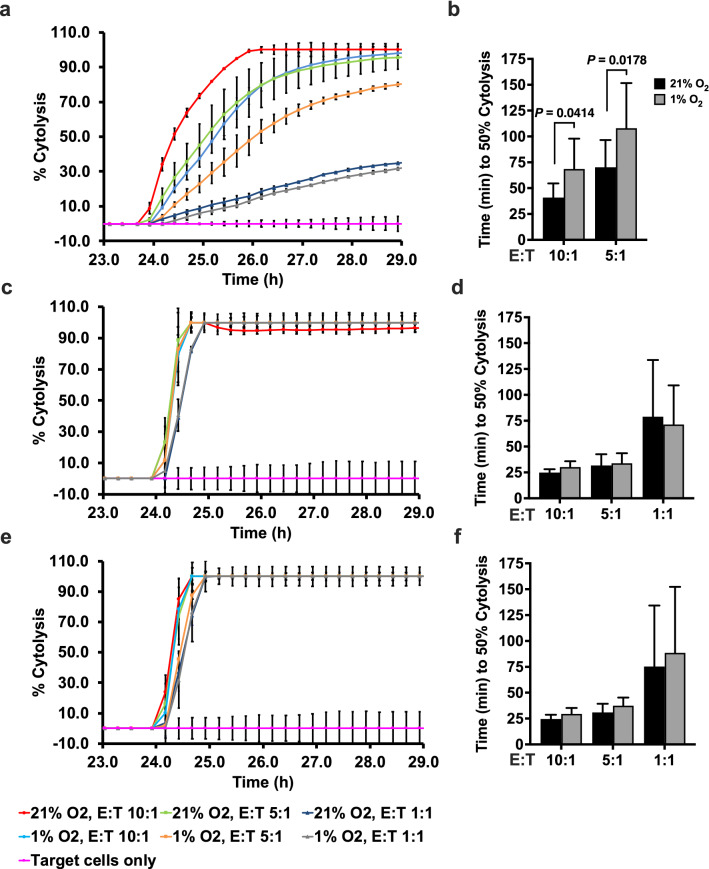
Real-time analysis of cytolysis exhibited by hypoxia-treated PBMC-derived NK cells against target tumor cells. Freshly isolated NK cells (**a**) or IL-2-expanded NK cells (**c**) or antibody-expanded NK cells (**e**) that were pre-incubated at 21% O_2_ or 1% O_2_ in the presence of IL-2 for 72 h were added to the target DLD-1 cells at E/T ratios of 10:1, 5:1, and 1:1. Real-time cytolysis of target cells was monitored using the xCELLigence RTCA SP system. Electrode impedance was measured and recorded as cell index. % Cytolysis was determined using the RTCA Software Pro. One representative of three independent experiments performed in duplicates using different donors is shown. **b**, **d**, and **f** show 50% killing time (KT 50) for the same E/T ratios in **a**, **c**, and **e**. *p ≤ *0.05 is significant

We next tested whether hypoxia had any functional effects on the PBMC-derived fresh and expanded NK cells. Freshly isolated NK cells were cultured under hypoxia or normoxia in the presence of IL-2 for a period of 72 h and subsequently co-cultured with DLD-1 cells and % cytotoxicity measured in real time. Target cells were killed by hypoxia pre-treated NK cells, although with a lower efficacy compared to normoxia-treated NK cells, as shown by the increased time required by hypoxia-treated NK cells to attain 50% cytolysis (E/T 10:1, *p* = 0.0414; E/T 5:1, *p* = 0.0178) (Fig. 7a and b). However, hypoxia-treated ex vivo expanded NK cells, irrespective of the method of expansion, did not show any significant differences in cytotoxicity compared to their normoxia-treated counterparts (Fig. 7c–f).

Thus, freshly isolated human NK cells which do not express HIF-1α showed an impaired cytotoxicity against tumor cells in hypoxia, while ex vivo expanded NK cells that express HIF-1α did not exhibit impaired cytotoxicity during hypoxia. Though hypoxic freshly isolated NK cells and ex vivo expanded NK cells showed reduction in expression of activation markers NKp46 and NKG2D, the simultaneous upregulation of CD69 in the ex vivo expanded NK could have compensated for any impairment of cytotoxicity due to the downregulation of these receptors on these cells. The influence of these activation receptors in determining NK cell cytotoxicity under hypoxic conditions needs to be further explored.

**Fig. 8:**
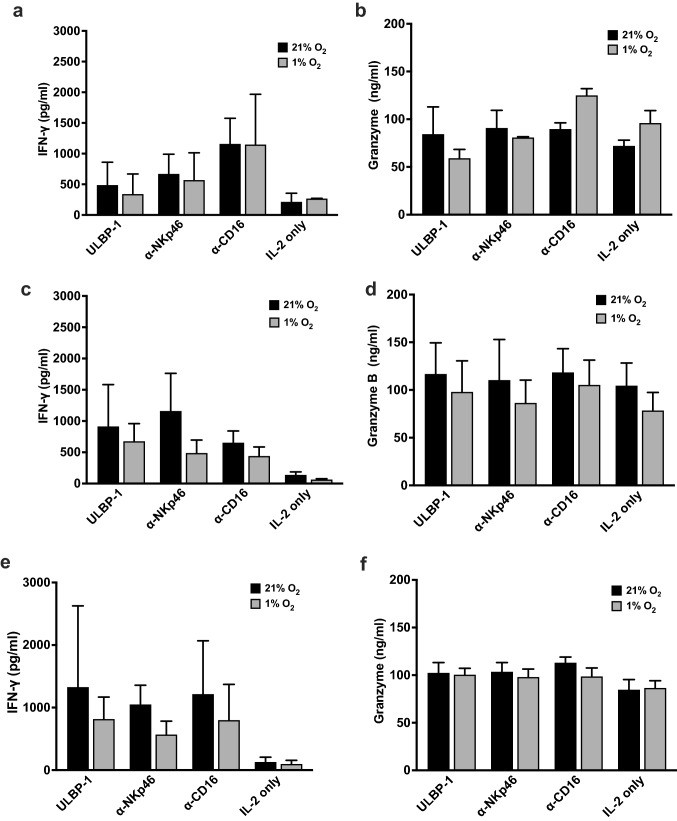
IFN-γ and granzyme B expression in hypoxia-treated PBMC-derived NK cells. Freshly isolated NK cells (**a**, **b**) or IL-2-expanded NK cells (**c**, **d**) or antibody-expanded NK cells (**e**, **f**) were pre-incubated at 21% O_2_ or 1% O_2_ in the presence of IL-2 for 72 h before being transferred to plates coated with ULBP-1 or anti-NKp46 or anti-CD16 antibodies and incubations continued under the same conditions for another 18 h. After incubation, supernatant was collected, and ELISA performed in duplicates to test the concentrations of IFN-γ and granzyme B. Results are presented as mean of three different donors. Statistical significance was analyzed between 21% O_2_ and 1% O_2_ by two-tailed paired Student’s *t*-test

We then measured the IFN-γ response of freshly isolated and expanded NK cells in normoxic and hypoxic conditions. Stimulation of freshly isolated NK cells with ULBP1 or with anti-CD16 and anti-NKp46 antibodies in hypoxia showed no significant changes in secretion of IFN-γ (Fig. 8a). However, the hypoxia-treated expanded NK cells showed a reduction in secretion of IFN-γ upon stimulation but this change was not significant within the donors we tested (Fig. 8c and e). On measuring expression of granzyme B, we observed no significant changes in its expression in either the freshly isolated or expanded NK cells when exposed to hypoxia (Fig. 8b, d, and f).

## Discussion

NK cells are innate immune cells that are capable of invading solid tumors, and a higher number of tumor-infiltrating NK cells are associated with better survival [19, 20]. However, NK cells that infiltrate the tumor bed are attenuated by factors within the TME. Hypoxia is one such factor in the TME that has been shown to modulate the functions of immune cells through expression of HIF-1α [21–23]. In this study, we investigated the mechanisms that regulate HIF-1α expression in NK cells and correlated HIF-1α expression with NK cell functions. Using the NK cell line, NKL, and ex vivo expanded NK cells, we demonstrate that HIF-1α expression requires both hypoxia and IL-2 stimulation. IL-2-mediated PI3K/mTOR signaling initiates HIF-1α protein synthesis, while hypoxia maintained its stabilization. Critically, we show that NKL cells expressing HIF-1α in hypoxia had a better ability to kill tumor cells after hypoxia exposure. In contrast, freshly isolated NK cells that could not stabilize HIF-1α showed an impaired cytotoxicity that was overcome upon their ex vivo expansion and HIF-1α stabilization.

IL-2 on engaging its receptor CD25 on NK cells stimulates PI3K and the downstream activation of mTOR [24, 25]. In our study, treatment with either the PI3K inhibitor LY294002 or mTOR inhibitor rapamycin resulted in inhibition of IL-2-mediated HIF-1α expression in NK cells, indicating that the activation of the PI3K/mTOR pathway is necessary for HIF-1α expression. The absence of an increase in HIF-1α transcript levels in NKL cells treated with IL-2 (irrespective of oxygen tension) and the complete absence of HIF-1α protein upon translational inhibition using cycloheximide suggests that IL-2 functions to regulate protein translation of HIF-1α to enable HIF-1α protein expression. Nevertheless, HIF-1α protein was degraded in IL-2-stimulated NKL cells in normoxia, in accordance with previously published findings by others demonstrating the role of hypoxia in HIF-1α stabilization [26]. Under normoxic conditions, HIF-1α is rendered unstable due to its hydroxylation and subsequent degradation by the proteasome, while in hypoxia, degradation is inhibited due to limited HIF-1α hydroxylation. In our experiments, IL-2-stimulated NKL cells treated with the proteasomal inhibitor MG132 showed a clear accumulation of HIF-1α under normoxic conditions, confirming the proteasome-dependent HIF-1α degradation during normoxia and the stabilizing role of hypoxia in maintaining HIF-1α expression levels in NKL cells.

On extending our observations from the NKL cell line to human PBMC-derived NK cells, we were surprised that NK cells freshly isolated from PBMCs of healthy human donors when cultured in hypoxia in the presence of IL-2 failed to show detectable HIF-1α protein. Interestingly, treating these cells with PHD inhibitor, DMOG, resulted in significant accumulation of HIF-1α protein in NK cells in both hypoxic and normoxic conditions. Thus, freshly isolated NK cells were capable of HIF-1α protein synthesis upon IL-2 stimulation but hypoxia failed to stabilize the protein against degradation. The ability of DMOG to prevent HIF-1α degradation and demonstrate detectable HIF-1α protein levels would indicate the continued activity of PHDs in freshly isolated NK cells even during hypoxia. One possibility for continued PHD activity could be explained by redistribution of cellular oxygen in the cytoplasm due to reduced mitochondrial respiration [27]. Assessing the metabolic status of freshly isolated NK cell could shed light on the lack of HIF-1α expression in these cells.

Ex vivo expansion of NK cells is an important source of NK cells for clinical application since it enables the efficient generation of large numbers of NK cells for repeated therapeutic use in cancer. Ex vivo expanded human NK cells are more cytotoxic and activated compared to freshly isolated NK cells [28]. Here, we demonstrate, for the first time, that ex vivo expanded NK cells, unlike freshly isolated NK cells, are capable of expressing HIF-1α under hypoxia upon IL-2 stimulation via the PI3K/mTOR pathway. The requirement of IL-2 signaling for HIF-1α protein synthesis in human NK cells that we have shown in this study is similar to signaling requirements for HIF-1α protein synthesis in T cells. Studies in CD4 + T cells have demonstrated that hypoxia alone was insufficient to induce HIF-1α protein, and a combination of TCR-mediated signal and hypoxia was required to induce HIF-1α accumulation [5, 29]. However, considering the pro-inflammatory environment in tumors, it is essential to thoroughly examine HIF-1α expression in NK cells, in the presence of other pro-inflammatory cytokines including IL-12, IL-18 and IL-21 that are known to play important roles in activating NK cells in the tumor microenvironment.

NK cells are early responders against tumors, a function mediated by two effector mechanisms—cytotoxicity and cytokine-secreting function. We specifically investigated whether expression of HIF-1α in the NKL cell line or the ex vivo expanded NK cells had any correlation with their anti-tumor cytotoxicity and cytokine-secreting functions. In hypoxia, NKL cells showed an increase in anti-tumor cytotoxicity as measured by the real-time cytotoxicity assay. Interestingly, they also secreted more IFN-γ when stimulated with ULBP-1 or anti-NKp46 or anti-CD16 antibodies. However, the enhanced cytotoxic response that we observed in hypoxic NKL cells could not be explained by the release of granzyme B or the expression of activating and inhibiting receptors which looked similar in both hypoxic and normoxic NKL cells. Examining the expression of a wider range of receptors including 2B4, CD94-NKG2C, NKp30, and NKp44 could shed light on the role of the receptors in NKL responses to hypoxia. Interestingly, we did not observe significant changes in secretion of granzyme B by hypoxic NK cells upon ligand stimulation. Though NK cells can kill target tumor cells by exocytosis of granzyme B, they also kill target cells by engaging death receptors such as FASL and TRAIL. The contribution of these receptors to cytotoxicity under hypoxia needs to be studied. Since HIF-1α mediates adaption to hypoxia by regulating cellular metabolism, it is probable that HIF-1α upregulation in hypoxic NKL cells may modulate its metabolism to alter effector functions.

Freshly isolated NK cells from human peripheral blood when pre-exposed to hypoxia showed a reduced ability to kill target tumor cells but no significant changes in secretion of IFN-γ, indicating that hypoxia dampened only the cytotoxicity functions. The impaired cytotoxicity by these cells could be explained by the reduction in expression of activation marker NKp46 which is a cytotoxicity triggering receptor demonstrated to be essential for cytotoxicity of freshly isolated NK cells [30]. It is of interest to note here that these effects are independent of HIF-1α since freshly isolated NK cells are unable to stabilize HIF-1α. Our observations here are different from the results reported in the study by Velasquez et al., where IL-15 stimulation of hypoxic NK cells did not significantly alter viability of target tumor cells any differently compared to IL-15-stimulated normoxic NK cells [31]. Though their method of hypoxic pre-culture of NK cells is comparable to ours, their conclusions were drawn from an end point cytotoxicity assay unlike the real-time quantification of cytolysis that we used in our study that provides significant and quantifiable cytotoxicity measurements at early time points.

In contrast to freshly isolated NK cells, hypoxia-treated ex vivo expanded NK cells, irrespective of the method of expansion, showed no impairment in cytotoxicity, despite the reduction in expression of activation markers NKG2D and NKp46. Interestingly, ex vivo expanded NK cells had a higher expression of CD69 in hypoxia, a stimulatory receptor for NK cell cytotoxicity, which could be responsible for overcoming any impairment of cytotoxicity during hypoxia [32].

An area of interest arising from our findings thus far is in identifying HIF-1α-mediated metabolic changes in NK cells. NK cells have uniquely different metabolic needs to execute their cytolytic and IFN-γ-secreting functions. Activation of NK cells with cytokines increases the rate of glucose-driven glycolysis and Oxphos that in turn drives cytotoxic functions, while they are capable of IFN-γ secretion in the absence of Oxphos [33–35]. The differential expression of HIF-1α in hypoxia could lead to distinct metabolic profiles of NKL, freshly isolated NK cells, and ex vivo expanded NK cells that could determine their functional differences in hypoxia, an area that remains to be investigated [36–38].

Adding credence to our observation that HIF-1α promotes cytotoxicity in hypoxic NK cells is a study in a mouse tumor model with a targeted deletion of HIF-1α in NK cells, showing an increase in tumor metastasis due to reduced cytotoxicity of NK cells lacking HIF-1α [39]. The lack of specific chemical inhibitors against HIF-1α expression has limited our ability to directly link the expression of HIF-1α to the enhanced cytotoxic effects we observed in the human NK cells in our experiments. However, genetic manipulation of HIF-1α in the NKL cell line in future experiments could provide direct evidence of effects of HIF-1α on human NK cell functions. Though in this study we have used the MHC-I-deficient colorectal tumor cell line DLD-1 to evaluate the effect of hypoxia on NK cell cytotoxicity, we have observed a similar increase in cytotoxicity of hypoxia-exposed NKL cells against an MHC-I-positive renal cell carcinoma cell line 786-O (data not shown), indicating that this effect is not restricted to MHC-I-deficient tumors. In addition, a relevant study by Sarkar et al. [40] has demonstrated that the expression of receptors for the human MHC-I antigens HLA-ABC, the killer immunoglobulin-like receptors (KIR), was not altered on NK cells upon exposure to hypoxia. Furthermore, we acknowledge that in this study we did not investigate the expression of HIF-2α in NK cells. HIF-2α is another important hypoxia-inducible factor expressed in T cell and macrophages that plays a prominent role in their function. Studying the expression of HIF-2α in NK cells and its effect on their function and investigating whether there is any crosstalk between HIF-1α and HIF-2α expression would elucidate cooperative and synergistic interactions between these transcription factors in modulating NK cell responses to hypoxia.

To our knowledge, this is for the first time that expression of HIF-1α in hypoxic human NK cells has been correlated with their function. Based on our study, it is likely that tumor-infiltrating NK cells that reach hypoxic tumor sites in the presence of IL-2 upregulate HIF to enhance NK cell function. Our discovery demonstrating enhanced effector functions in hypoxia pretreated NKL cells and ex vivo expanded NK cells strengthens their potential use as effective candidates for adoptive immunotherapy.

## Supplementary Information

Below is the link to the electronic supplementary material.

Supplementary file1 (PDF 2203 KB)

## References

[1] Sivori S, Vacca P, Del Zotto G, Munari E, Mingari MC, Moretta L (2019). Human NK cells: surface receptors, inhibitory checkpoints, and translational applications. Cell Mol Immunol.

[2] Fink T, Ebbesen P, Koppelhus U, Zachar V (2003). Natural killer cell-mediated basal and interferon-enhanced cytotoxicity against liver cancer cells is significantly impaired under in vivo oxygen conditions. Scand J Immunol.

[3] Hudson CC, Liu M, Chiang GG, Otterness DM, Loomis DC, Kaper F, Giaccia AJ, Abraham RT (2002). Regulation of hypoxia-inducible factor 1α expression and function by the mammalian target of rapamycin. In Mol Cell Biol.

[4] Wang GL, Jiang B-H, Rue EA, Semenza GL (1995) Hypoxia-inducible factor 1 is a basic-helix-loop-helix-PAS heterodimer regulated by cellular 02 tension. Proc Natl Acad Sci USA 5 10.1073/pnas.92.12.5510 PMC41725 7539918

[5] Nakamura H, Makino Y, Okamoto K, Poellinger L, Ohnuma K, Morimoto C, Tanaka H (2005). TCR engagement increases hypoxia-inducible factor-1 alpha protein synthesis via rapamycin-sensitive pathway under hypoxic conditions in human peripheral T cells. J Immunol.

[6] Dang EV, Barbi J, Yang HY, Jinasena D, Yu H, Zheng Y, Bordman Z, Fu J, Kim Y, Yen HR (2011). Control of T(H)17/T(reg) balance by hypoxia-inducible factor 1. Cell.

[7] Blouin CC, Page EL, Soucy GM, Richard DE (2004). Hypoxic gene activation by lipopolysaccharide in macrophages: implication of hypoxia-inducible factor 1alpha. Blood.

[8] Joshi S, Singh AR, Zulcic M, Durden DL (2014). A macrophage-dominant PI3K isoform controls hypoxia-induced HIF1alpha and HIF2alpha stability and tumor growth, angiogenesis, and metastasis. Mol Cancer Res.

[9] Jensen H, Potempa M, Gotthardt D, Lanier LL (2017). Cutting edge: IL-2-induced expression of the amino acid transporters SLC1A5 and CD98 Is a prerequisite for NKG2D-mediated activation of human NK cells. J Immunol.

[10] de Rham C, Ferrari-Lacraz S, Jendly S, Schneiter G, Dayer JM, Villard J (2007). The proinflammatory cytokines IL-2, IL-15 and IL-21 modulate the repertoire of mature human natural killer cell receptors. Arthritis Res Ther.

[11] Lee J, Fenton BM, Koch CJ, Frelinger JG, Lord EM (1998). Interleukin 2 expression by tumor cells alters both the immune response and the tumor microenvironment. Can Res.

[12] Law TM, Motzer RJ, Mazumdar M, Sell KW, Walther PJ, O'Connell M, Khan A, Vlamis V, Vogelzang NJ, Bajorin DF (1995). Phase III randomized trial of interleukin-2 with or without lymphokine-activated killer cells in the treatment of patients with advanced renal cell carcinoma. Cancer.

[13] Kedia-Mehta N, Choi C, McCrudden A, Littwitz-Salomon E, Fox PG, Gardiner CM, Finlay DK (2019) Natural killer cells integrate signals received from tumour interactions and IL2 to induce robust and prolonged anti-tumour and metabolic responses. Immunometabolism 1:e190014 10.20900/immunometab20190014 PMC6783304 31595191

[14] Magdaleno C, Dixon L, Rajasekaran N, Varadaraj A (2020). HIFalpha independent mechanisms in renal carcinoma cells modulate divergent outcomes in fibronectin assembly mediated by hypoxia and CoCl2. Sci Rep.

[15] Wu Y, Tian Z, Wei H (2017). Developmental and functional control of natural killer cells by cytokines. Front Immunol.

[16] Marcais A, Marotel M, Degouve S, Koenig A, Fauteux-Daniel S, Drouillard A, Schlums H, Viel S, Besson L, Allatif O et al (2017) High mTOR activity is a hallmark of reactive natural killer cells and amplifies early signaling through activating receptors. Elife 6 10.7554/eLife.26423 PMC5628014 28875936

[17] Park KU, Jin P, Sabatino M, Feng J, Civini S, Khuu H, Berg M, Childs R, Stroncek D (2010). Gene expression analysis of ex vivo expanded and freshly isolated NK cells from cancer patients. J Immunother.

[18] Masuyama J, Murakami T, Iwamoto S, Fujita S (2016). Ex vivo expansion of natural killer cells from human peripheral blood mononuclear cells co-stimulated with anti-CD3 and anti-CD52 monoclonal antibodies. Cytotherapy.

[19] Zhang S, Liu W, Hu B, Wang P, Lv X, Chen S, Shao Z (2020). Prognostic significance of tumor-infiltrating natural killer cells in solid tumors: a systematic review and meta-analysis. Front Immunol.

[20] Cursons J, Souza-Fonseca-Guimaraes F, Foroutan M, Anderson A, Hollande F, Hediyeh-Zadeh S, Behren A, Huntington ND, Davis MJ (2019). A Gene signature predicting natural killer cell infiltration and improved survival in melanoma patients. Cancer Immunol Res.

[21] Westendorf AM, Skibbe K, Adamczyk A, Buer J, Geffers R, Hansen W, Pastille E, Jendrossek V (2017). Hypoxia enhances immunosuppression by inhibiting CD4+ effector T cell function and promoting Treg activity. Cell Physiol Biochem.

[22] Leblond MM, Gerault AN, Corroyer-Dulmont A, MacKenzie ET, Petit E, Bernaudin M, Valable S (2016) Hypoxia induces macrophage polarization and re-education toward an M2 phenotype in U87 and U251 glioblastoma models. Oncoimmunology 5:e1056442 10.1080/2162402X.2015.1056442 PMC4760330 26942063

[23] Pietrobon V, Marincola FM (2021). Hypoxia and the phenomenon of immune exclusion. J Transl Med.

[24] Kuo CJ, Chung J, Fiorentino DF, Flanagan WM, Blenis J, Crabtree GR (1992). Rapamycin selectively inhibits interleukin-2 activation of p70 S6 kinase. Nature.

[25] Reif K, Burgering BM, Cantrell DA (1997). Phosphatidylinositol 3-kinase links the interleukin-2 receptor to protein kinase B and p70 S6 kinase. J Biol Chem.

[26] Semenza GL (2004). Hydroxylation of HIF-1: oxygen sensing at the molecular level. Physiology (Bethesda).

[27] Hagen T, Taylor CT, Lam F, Moncada S (2003). Redistribution of intracellular oxygen in hypoxia by nitric oxide: effect on HIF1alpha. Science.

[28] Nham T, Poznanski SM, Fan IY, Shenouda MM, Chew MV, Lee AJ, Vahedi F, Karimi Y, Butcher M, Lee DA (2018). Ex vivo-expanded NK cells from blood and ascites of ovarian cancer patients are cytotoxic against autologous primary ovarian cancer cells. Cancer Immunol immunother CII.

[29] Palazon A, Tyrakis PA, Macias D, Velica P, Rundqvist H, Fitzpatrick S, Vojnovic N, Phan AT, Loman N, Hedenfalk I et al (2017) An HIF-1alpha/VEGF-A axis in cytotoxic T cells regulates tumor progression. Cancer Cell 32, 669–683 e665 10.1016/j.ccell.2017.10.003 PMC5691891 29136509

[30] Sivori S, Pende D, Bottino C, Marcenaro E, Pessino A, Biassoni R, Moretta L, Moretta A (1999). NKp46 is the major triggering receptor involved in the natural cytotoxicity of fresh or cultured human NK cells. Correlation between surface density of NKp46 and natural cytotoxicity against autologous, allogeneic or xenogeneic target cells. Eur J Immunol.

[31] Velasquez SY, Killian D, Schulte J, Sticht C, Thiel M, Lindner HA (2016). Short Term hypoxia synergizes with interleukin 15 priming in driving glycolytic gene transcription and supports human natural killer cell activities. J Biol Chem.

[32] Borrego F, Robertson MJ, Ritz J, Pena J, Solana R (1999). CD69 is a stimulatory receptor for natural killer cell and its cytotoxic effect is blocked by CD94 inhibitory receptor. Immunology.

[33] Keppel MP, Saucier N, Mah AY, Vogel TP, Cooper MA (2015). Activation-specific metabolic requirements for NK Cell IFN-gamma production. J Immunol.

[34] Mah AY, Cooper MA (2016). Metabolic regulation of natural killer cell IFN-gamma production. Crit Rev Immunol.

[35] Mah AY, Rashidi A, Keppel MP, Saucier N, Moore EK, Alinger JB, Tripathy SK, Agarwal SK, Jeng EK, Wong HC et al (2017) Glycolytic requirement for NK cell cytotoxicity and cytomegalovirus control. JCI Insight 2 10.1172/jci.insight.95128 PMC5752285 29212951

[36] Papandreou I, Cairns RA, Fontana L, Lim AL, Denko NC (2006). HIF-1 mediates adaptation to hypoxia by actively downregulating mitochondrial oxygen consumption. Cell Metab.

[37] Zhang H, Bosch-Marce M, Shimoda LA, Tan YS, Baek JH, Wesley JB, Gonzalez FJ, Semenza GL (2008). Mitochondrial autophagy is an HIF-1-dependent adaptive metabolic response to hypoxia. J Biol Chem.

[38] Nagao A, Kobayashi M, Koyasu S, Chow CCT, Harada H (2019) HIF-1-Dependent reprogramming of glucose metabolic pathway of cancer cells and its therapeutic significance. Int J Mol Sci 20 10.3390/ijms20020238 PMC6359724 30634433

[39] Krzywinska E, Kantari-Mimoun C, Kerdiles Y, Sobecki M, Isagawa T, Gotthardt D, Castells M, Haubold J, Millien C, Viel T (2017). Loss of HIF-1alpha in natural killer cells inhibits tumour growth by stimulating non-productive angiogenesis. Nat Commun.

[40] Sarkar S, Germeraad WT, Rouschop KM, Steeghs EM, van Gelder M, Bos GM, Wieten L (2013). Hypoxia induced impairment of NK cell cytotoxicity against multiple myeloma can be overcome by IL-2 activation of the NK cells. PLoS ONE.

