# Supplementary material for: Hypoxia-inducible factor-1 alpha expression is induced by IL-2 via the PI3K/mTOR pathway in hypoxic NK cells and supports effector functions in NKL cells and ex vivo expanded NK cells
Source: Cancer Immunol Immunother. 2022 Jan 9;71(8):1989–2005. doi: 10.1007/s00262-021-03126-9 (PMC9294031; doi:10.1007/s00262-021-03126-9)
Supplement: Supplementary file 1 — Supplementary file1 (PDF 2203 KB) [file 262_2021_3126_MOESM1_ESM.pdf]

Supplementary Table 1

**List of antibodies**			
**Antibody**	**Clone**	**Source**	**Catalog#**
Flowcytometry			
NKp46	9E2	Biolegend	331908
CD16	3G8	Biolegend	302026
NKG2D	1D11	Biolegend	320818
NKG2A	S1940004C	Biolegend	375108
CD69	FN50	Biolegend	310903
CD56	HCD56	Biolegend	318310
Immunoblotting			
Hif-1α	D2U3T	Cell Signaling	14179
p70 S6 Kinase	Polyclonal	Cell Signaling	9202
Phospho-p70 S6 Kinase (Thr389)	Polyclonal	Cell Signaling	9205
β- Actin	15G5A11/E2	ThermoFisher Scientific	MA1-140
Anti-ERK1 + ERK2 antibody	Polyclonal	Abcam	ab17942
Anti-ERK1 + ERK2 (phospho T202 + Y204)	Polyclonal	Abcam	ab214362
ELISA			
UltraLeaf anti-NKp46 ab	9E2	Biolegend	331947
UltraLeaf anti-CD16 ab	3G8	Biolegend	302057

Supplementary Fig.1

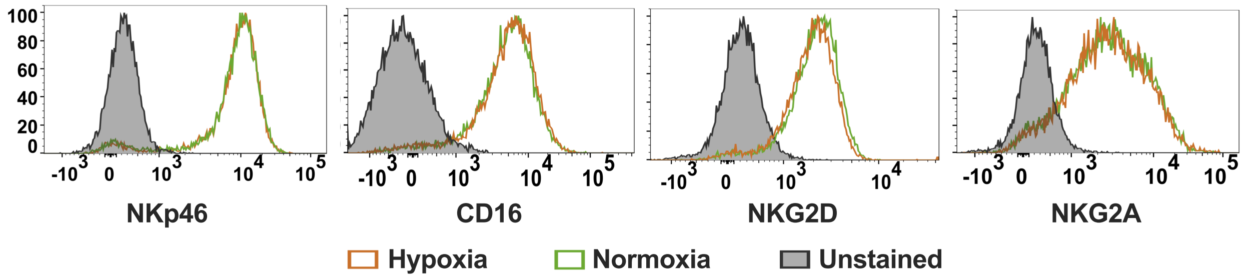

Characterization of NK cell receptors on NKL cells in hypoxia

NKL cells in presence of IL-2 were exposed to 21% or 1% O_2 for 24 h. Expression of surface receptors NKG2D, NKp46, CD16 and NKG2A was analyzed by flowcytometry. Representative histograms of expression of these receptors on NKL cells is shown.

Supplementary Fig.2

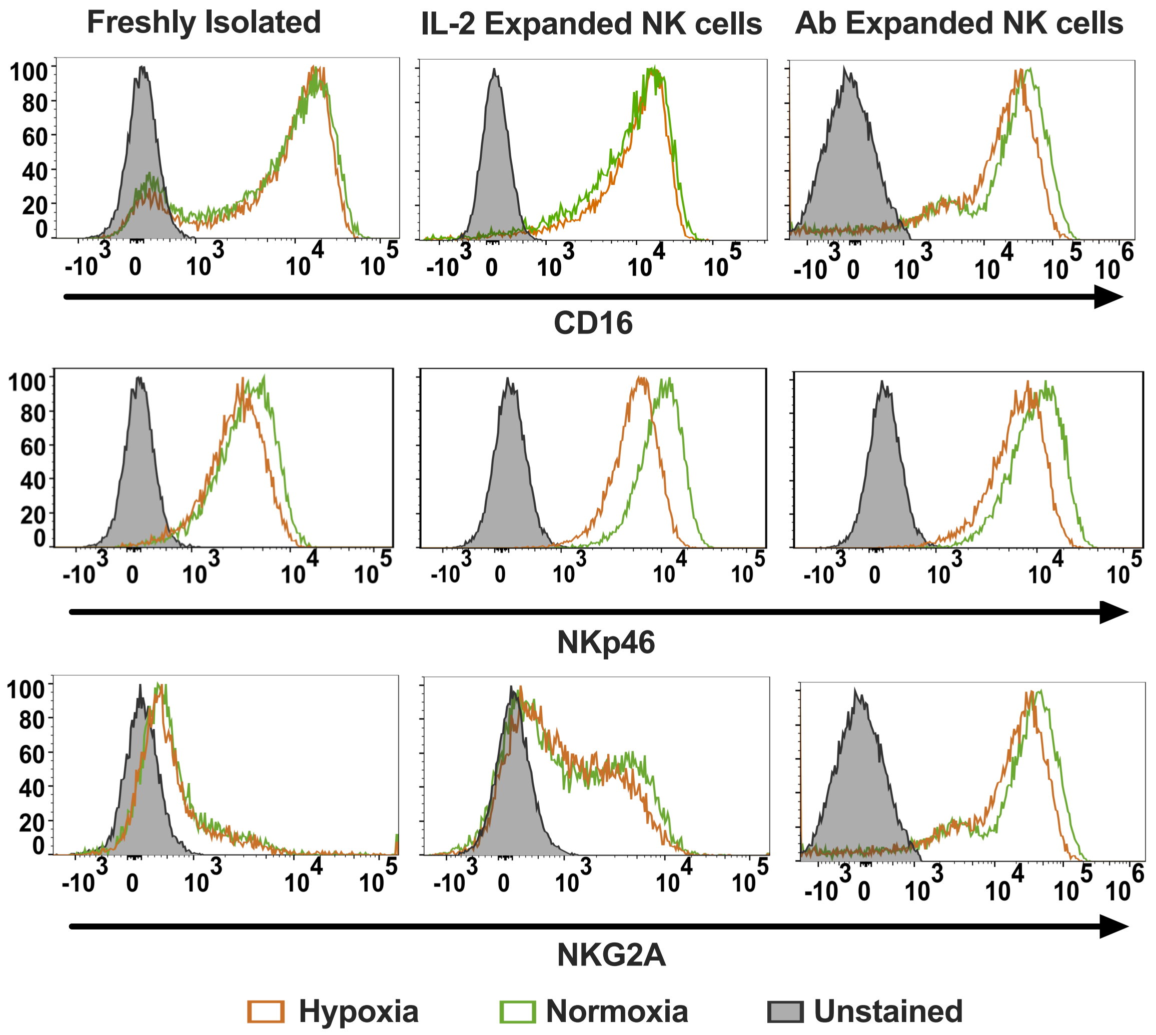

Characterization of NK cell receptors on PBMC derived NK cells in hypoxia

Freshly isolated NK cells and ex vivo expanded NK cells were stimulated with IL-2 and incubated in 21% O_2 or 1% O_2 for 72 h. Expression of surface receptors was analyzed by flowcytometry as described in the materials and methods. Representative histograms of CD16, NKp46 and NKG2A expression on freshly isolated NK cells, IL-2 expanded NK cells and antibody expanded NK cells are shown.
